# Development of a multienzyme isothermal rapid amplification and lateral flow dipstick combination assay for bovine coronavirus detection

**DOI:** 10.3389/fvets.2022.1059934

**Published:** 2023-01-04

**Authors:** Chengyuan Ji, Yiqiu Feng, Ruini Sun, Qibing Gu, Yao Zhang, Jiale Ma, Zihao Pan, Huochun Yao

**Affiliations:** MOE Joint International Research Laboratory of Animal Health and Food Safety, College of Veterinary Medicine, Nanjing Agricultural University, Nanjing, China

**Keywords:** bovine coronavirus, detection, multienzyme isothermal rapid amplification, lateral flow dipstick, diagnostic

## Abstract

Bovine coronavirus (BCoV) is a major cause of infectious disease in cattle, causing huge economic losses to the beef and dairy industries worldwide. BCoV can infect humans and multiple other species of animals. A rapid, reliable, and simple test is needed to detect BCoV infection in suspected farms. In this study, we developed a novel multienzyme isothermal rapid amplification (MIRA) and lateral flow dipstick (LFD) combination assay, targeting a highly conserved region of the viral nucleocapsid (*N*) gene for BCoV detection. The MIRA-LFD assay was highly specific and sensitive, comparable to a published reverse transcription quantitative PCR (RT-qPCR) assay for BCoV detection. Compared with the published RT-qPCR assay, the κ value of the MIRA-LFD assay in the detection of 192 cattle clinical samples was 0.982. The MIRA-LFD assay did not require sophisticated instruments and the results could be observed with eyes. Our results showed that the MIRA-LFD assay was a useful diagnostic tool for rapid on-site detection of BCoV.

## Introduction

Coronaviruses are lipid-enveloped and include severe acute respiratory syndrome-associated coronavirus, severe acute respiratory syndrome coronavirus 2 (SARS-CoV-2), and Middle East respiratory syndrome-associated coronavirus. They exhibit variable tissue tropism and the ability to infect various species of hosts, as exemplified by SARS-CoV-2 (1). Bovine coronavirus (BCoV) belongs to the genus *Betacoronavirus* of the family *Coronaviridae*, with a single-stranded and positive-sense RNA genome (2). BCoV has been linked with several clinical syndromes, including neonatal calf diarrhea (3, 4), winter dysentery (5), bovine respiratory disease, and shipping fever in adult cattle (6–8). The morbidity of BCoV ranged from 2 to 84%, and the mortality varied among cattle of different ages, and was approximately 8% in cattle with 5–90 days old (9, 10). BCoV is prevalent worldwide and spreads *via* the fecal-oral and respiratory routes (11, 12). Infections of BCoV and BCoV-like viruses have been found in goats, dromedary camels, deers, dogs, and humans (13–16).

Several diagnostic methods have been developed for detecting and identifying BCoV infections, including direct electron microscopy, protein A-gold immunoelectron microscopy (17), hybridization with cDNA probes (18), direct immunofluorescence (19), immunofluorescent staining, immune electron microscopy (20), immunohistochemistry (21), indirect ELISA (22), DNA microarray (23), rapid immunochromatographic assay (24), reverse transcription-polymerase chain reaction (RT-PCR) (25), semi-nested PCR assay (26), multiplex RT-PCR (27), and quantitative RT-PCR (RT-qPCR) (28, 29). These assays play a vital role in the detection of BCoV infections, but their applications are restricted because they require trained technicians, complex and expensive instruments, and/or long turn-over time. Therefore, these methods are unsuitable to be used at farms. In this study, we aimed to develop a rapid, reliable, simple, and isothermal amplification-based assay to fill the technological gap in BCoV detection at farms.

**Figure 1:**
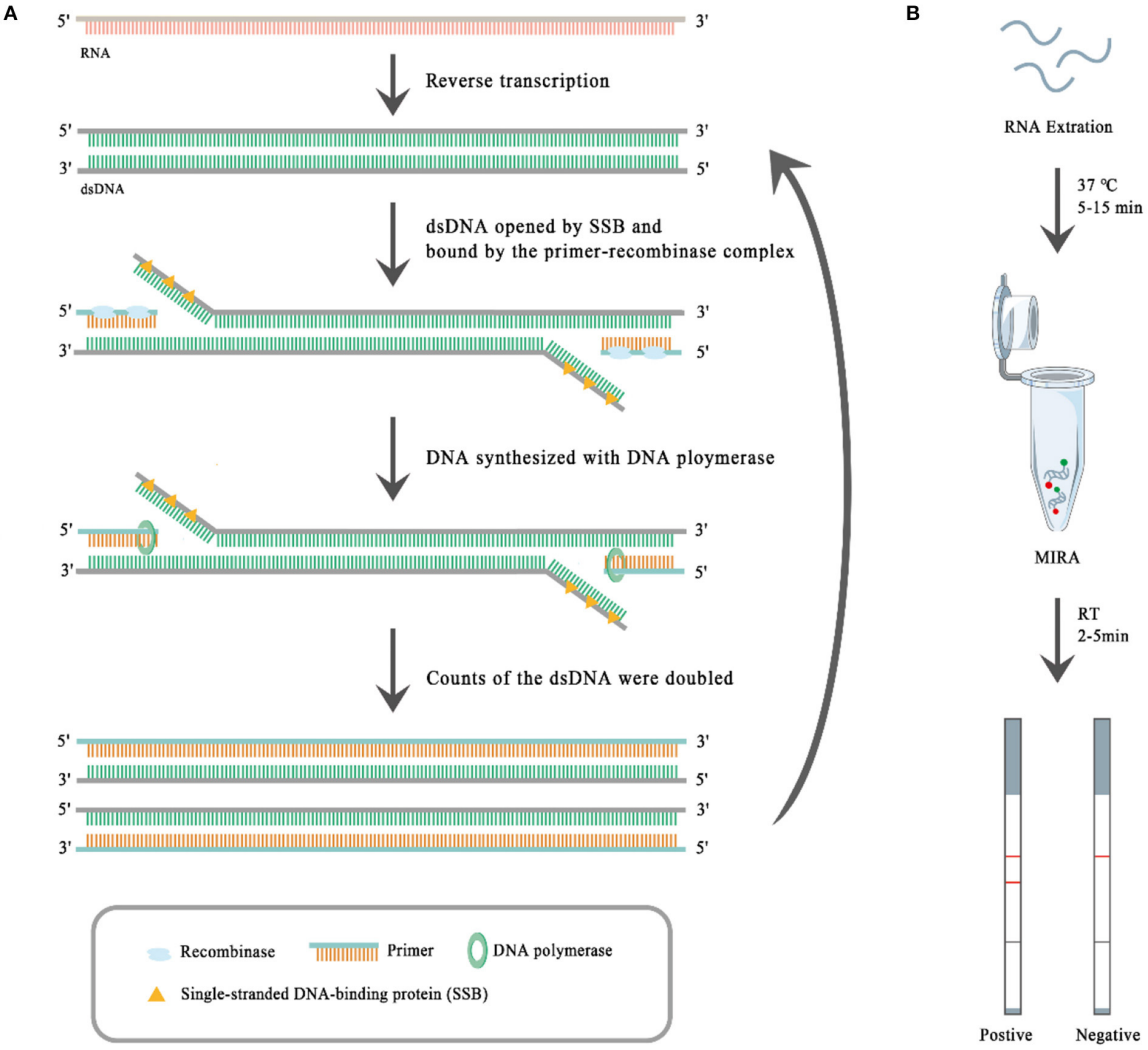
The principle of multienzyme isothermal rapid amplification (MIRA) **(A)** and the workflow of the MIRA-lateral flow dipstick (MIRA-LFD) assay **(B)**. The presence of two red lines indicates a positive result and one red line a negative result.

To date, several isothermal amplification-based techniques, such as the loop-mediated isothermal amplification assay (LAMP), recombinase polymerase amplification (RPA), and multienzyme isothermal rapid amplification (MIRA) (30, 31) have been developed for detecting pathogens. MIRA, whose principle is similar to that of RPA, is a new rapid isothermal amplification technique for the detection of nucleic acids. As shown in Figure 1A, at the first step of MIRA reverse transcriptase (moloney murine leukemia virus reverse transcriptase, M-MLV RT) reversely transcribes RNA into dsDNA. Then the complex of recombinase (the product of the recA locus in *E. coli*) and the primer binds to the dsDNA, which has been opened by single-stranded DNA-binding (SSB) protein (i.e., T4 phage gene 32 protein, gp32). After the binding, the recombinase is removed and the DNA polymerase (*E. coli* DNA polymerase I) binds to the 3' end of the primer and catalyzes the chain extension, which doubles the dsDNA. The synthesized dsDNA shall be opened by SSB and bound by the complex of recombinase and the primer, which begets a new round of dsDNA synthesis. The MIRA reaction can be completed within 20 min at 35–42°C (30, 31). It is fast and sensitive, and can be combined with a lateral flow dipstick (LFD) to visually evaluate the results (Figure 1B). The results of MIRA can be read out with the LFD step, which, like MIRA, does not require expensive instruments or trained technicians. The target RNA is amplified using biotin-labeled primers through MIRA, and the specific amplicons are bound by specific FAM-labeled probes, so the specific amplicons carry both FAM and biotin haptens. When the specific amplicons move from one terminal to another terminal of the test strip, the specific amplicons bind through their FAM haptens to FAM-antibodies conjugated with golden nanoparticles, and then the specific amplicons bind through their biotin haptens to the T-line where the biotin-antibodies have been immobilized, so the golden nanoparticles show red color there. The remaining FAM-antibodies conjugated with golden nanoparticles bind to the control (C)-line where the secondary antibodies against the FAM-antibodies have been immobilized, and so the golden nanoparticles show red color there. Therefore, red T-lines suggest positive MIRA results, and red C-lines suggest the FAM-antibodies conjugated with golden nanoparticles work well. Therefore, the MIRA-LFD combination assay has obvious advantages for virus detection, particularly at farms with limited resources. In this study, we developed a MIRA-LFD combination assay for BCoV detection and evaluated its sensitivity and specificity.

## 2. Materials and methods

### 2.1. Viruses and plasmids

Field strains of bovine enterovirus (BEV), bovine herpesvirus-1 (BHV-1), bovine viral diarrhea virus 1 (BVDV-1) were cultured using MDBK (Madin-Darby bovine kidney) cells. Bovine clinical samples previously detected by RT-PCR and sequencing as positive in the detection of BCoV, bovine astrovirus (BAstV), or bovine rotavirus (BRV) were stored in our laboratory. The BCoV N gene was cloned into the pUC57 plasmid by Tsingke Biotechnology Company (Beijing, China).

### 2.2. Design and synthesis of primers and probes

To obtain the best diagnostic target for BCoV, we compared the full-length N gene sequences of BCoVs available in the GenBank. The N-terminal region of the gene is highly conserved and was hence selected as the target for the MIRA-LFD combination assay. The MIRA primers targeting the BCoV N gene were designed following the guidelines of AMP-Future Biotech company (Weifang, China). The sequences of the MIRA primers and the primers and probe of a RT-qPCR assay for BCoV detection, which was reported previously (28), were listed in Table 1. They were synthesized by Sangon Biotech Company (Shanghai, China) and purified with high-performance liquid chromatography (HPLC).

**Table 1:** The primers and probes used in this study.

**Primer**	**Sequence (5^′^-3^′^)**	**Source**
N-1F	CTACTGAAGCTAAAGGGTACTGGTACAGAC	This study
N-1R	[5′Biotin]CCTTCAATATAGTAACCCTGAGGGAGTACC	This study
N-2F^a^	CTACTGAAGCTAAAGGGTACTGGTACAGAC	This study
N-2R	[5′Biotin]GTATTGACATCAGCCTGGTTACTAGCGACC	This study
N-3F	AAAGGGTACTGGTACAGACACAACAGACGTTCC	This study
N-3R	[5′Biotin]GTCAGCCGGGGTATTGACATCAGCCTGGTTACT	This study
BCoV-Probe	[5′FAM]TTACTATCTTGGAACAGGACCGCATGCCAA[THF]GACCAGTATGGCACC[3′C3-Spacer]	This study
BCoV-qF	CTGGAAGTTGGTGGAGTT	(26)
BCoV-qR	ATTATCGGCCTAACATACATC	(26)
BCoV-qProbe^b^	[5′FAM]CCTTCATATCTATACACATCAAGTTGTT[3′BHQ1]	(26)

a BCoV-2F primer is same as BCoV-1F.

b FAM, thymidine nucleotide carrying fluorescein; THF: tetrahydrofuran spacer; C3-Spacer, C3 spacer at the 3′ end to block elongation; BHQ1, thymidine nucleotide carrying Black Hole Quencher 1.

### 2.3. Extraction of RNA and DNA

Each feces or swab sample was homogenized in fivefold volume of phosphate-buffered saline (PBS) (pH 7.2). Viral DNA/RNA was extracted using the Vazyme FastPure Viral DNA/RNA Mini Kit from Vazyme Company (Nanjing, China) from 200 μL of the virus-infected cell culture supernatant or the homogenized clinical samples. The extracted DNA/RNA was dissolved in 50 μL nuclease-free water and stored at −80°C until the relevant assays were performed.

### 2.4. The MIRA reactions

The MIRA reactions were performed using the AMP-Future Biotech company kit (#WLRB8204KIT), according to the manufacturer's instructions. The reaction mixture contained 10 μL A buffer, 4 μL ddH_2_O, 1 μL of each primer (10 μM) 2 μL B buffer, and 2 μL RNA template. The mixture was incubated in a 42°C water bath for 30 min, after which, each mixture (5 μL) was subjected to electrophoresis with a 2% (g/g) agarose gel for 10 min at 160 V. Finally, the samples were visualized using an automated digital gel image analysis system (Bio-Rad).

### 2.5. The MIRA-LFD assay

MIRA-LFD reactions were performed using a kit (# WLRN8206KIT) from AMP-Future Biotech Company according to the manufacturer's instructions. The reaction mixture contained 29.4 μL A buffer, 11.5 μL ddH_2_O, 2 μL of each primer (10 μM), 2.5 μL B buffer, 0.6 μL probe, and 2 μL RNA template. After the 10 min reaction in a water bath at 37–39°C, the product was 1:10,000 diluted with H_2_O. The diluted product (100 μL) was added to the LFD strip (Gu'An Beiji Biotech Company, Hebei, China), and the color was observed 5 min later.

### 2.6. The specificity evaluation

The RNA of Nucleic acid templates prepared from other bovine pathogens, including BVDV-1, BHV-1, BRV, BEV, and BAstV, were detected using the MIRA-LFD assay to evaluate the specificity of this assay.

### 2.7. The sensitivity evaluation

To determine the analytical sensitivity of the MIRA-LFD assay, the BCoV-N-pUC57 plasmid was diluted to 10^1^-10^8^ copies per reaction and detected with the MIRA-LFD assay with three replicates for each dilution, using ribozyme-free water as the negative control. The extracted RNA of three BCoV positive samples was diluted serially from 1:5, and the diluted RNA was detected with the MIRA-LFD assay and the RT-qPCR assay reported previously (28).

### 2.8. Evaluation with clinical samples

A total of 132 feces samples and 60 nasal swab samples of bovine clinical samples were collected from cattle in Hebei, Inner Mongolia, Heilongjiang, and Ningxia provinces or autonomous regions of China between 2021 and 2022. The samples were collected and submitted to our laboratory by the relevant animal owners. These samples were detected in triplicate with the MIRA-LFD assay and the RT-qPCR assay reported previously (28).

### 2.9. Statistical analyses

The Kappa (κ) and possibility (*p*) values were used to compare the detection results of the MIRA-LFD assay and the RT-qPCR assay. The confidence level for statistical analyses was set as 95% (*p* < 0.05). Statistical analysis was performed using SPSS (version 21.0).

## 3. Results

### 3.1. Selection of the primer pair and the reaction temperature

**Figure 2:**
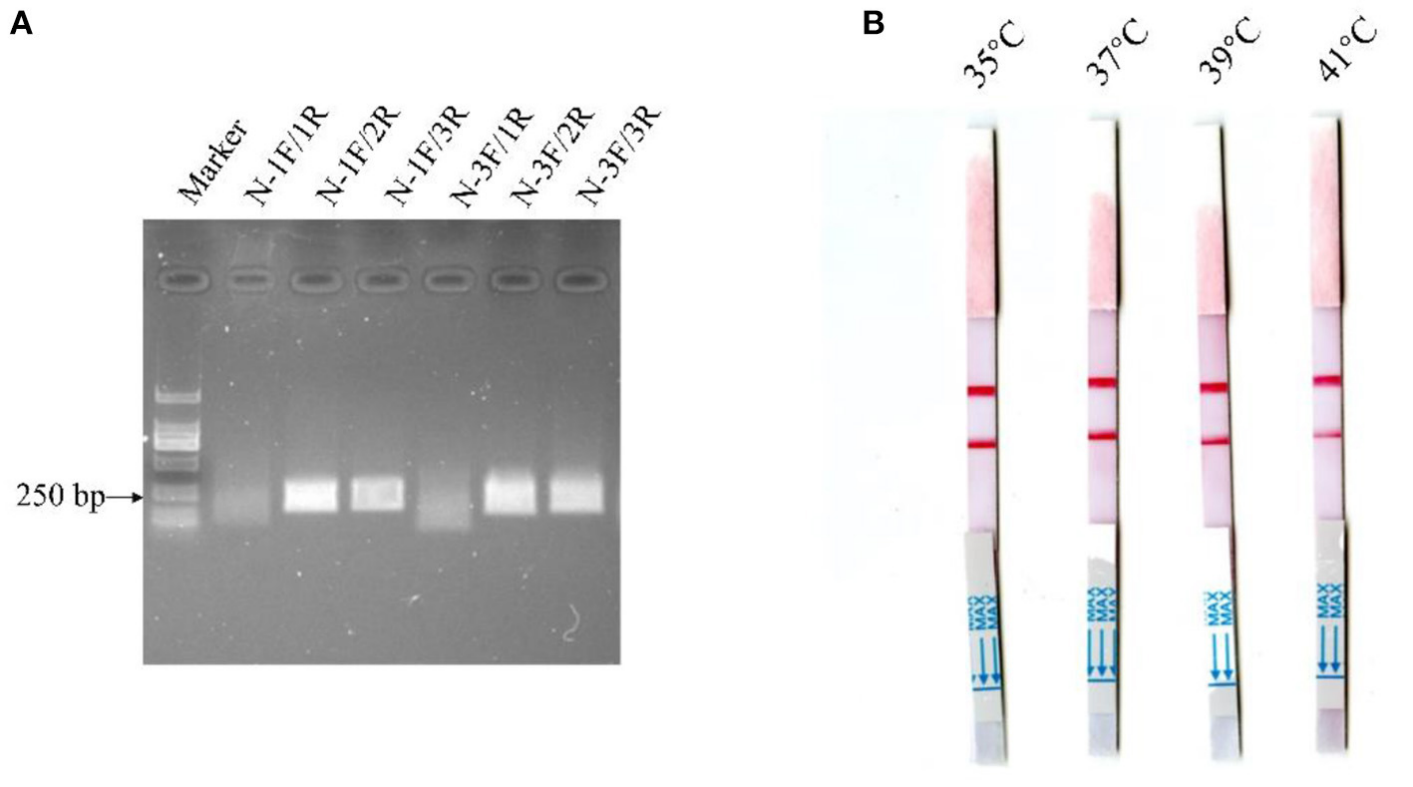
Detection of the diluted BCoV-N-pUC57 plasmid with the MIRA reactions using six pairs of primers **(A)** and comparison of the reaction temperatures for the MIRA-LFD assay **(B)**.

Alignment of some representative BCoV genome sequences suggested that the viral N gene sequences were relatively conserved, and hence the gene was selected as the target gene for the MIRA-LFD assay. Then, a suitable fluorescent N-Probe was designed for the assay, and two forward primers (N-1F, N-3F) and three reverse primers (N-1R, N-2R, and N-3R) were designed to match N-Probe. The MIRA reactions were performed to compare six combinations of the upstream and downstream primers using 10^4^ copies of the BCoV-N-pUC57 plasmid. As shown in Figure 2A, the combination of N-1F and N-2R was rated as one of the best-performing primer pairs and hence employed for the MIRA-LFD assay.

The MIRA-LFD assay was performed at different temperatures (35, 37, 39, and 41°C) using the manufacturer's instructions. As shown in Figure 2B, the red line was the strongest and was produced in the shortest time at 37°C, which was therefore selected as the reaction temperature for the MIRA-LFD assay.

### 3.2. Evaluation with known viruses or controls

**Figure 3:**
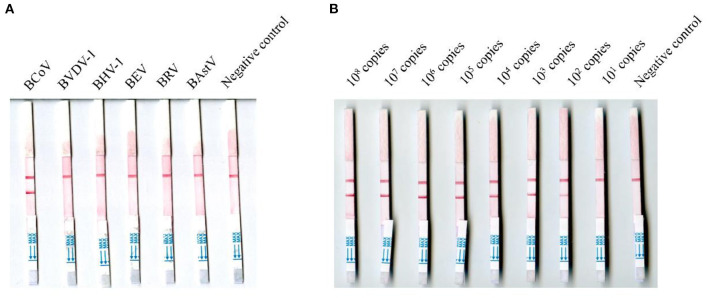
Detection of the RNA of six bovine viruses and the negative control **(A)** and different copies of the BCoV-N-pUC57 plasmid using the MIRA-LFD assay **(B)**.

The viral RNA molecules of BCoV, BVDV-1, BHV-1, BRV, BEV, and BAstV were tested using the MIRA-LFD assay, and none of them were positive except the viral RNA molecules of BCoV (Figure 3A). The BCoV-N-pUC57 plasmid was tested using the MIRA-LFD assay after serial dilution, and Figure 3B showed that the assay could detect 100 copies of the plasmid. The RT-qPCR could detect 100 copies of the plasmid as well. The extracted RNA of three BCoV positive samples (samples 21, 46, and 78) was diluted serially from 1:5, and both the MIRA-LFD and the RT-qPCR assay could detect the RNA after 1:5 dilution (samples 21 and 78) or 1:25 dilution (sample 46).

### 3.3. Evaluation with clinical samples

We detected 192 clinical samples submitted from several provinces or autonomous regions in China using the MIRA-LFD assay and the RT-qPCR assay reported previously. The RT-qPCR assay identified 35 (18.23%) BCoV positive samples, of which 34 were detected positive by the MIRA-LFD assay. The RT-qPCR assay identified 157 (81.77%) BCoV negative samples, which were all detected negative by the MIRA-LFD assay. The results of these two assays were highly consistent with each other (κ = 0.982) (Table 2).

**Table 2:** Detection of BCoV in clinical samples using two assays.

	**MIRA-LFD positive**	**MIRA-LFD negative**	**Kappa (κ)**
RT-qPCR positive	34	1	0.982
RT-qPCR negative	0	157	
Total	34	158	

## 4. Discussion

BcoV causes significant economic losses in the beef and dairy industries worldwide and can infect humans and multiple other species of animals (32). Rapid and accurate detection of BCoV is vital for the control of the virus infection. In this study, we established a new rapid assay for the BCoV detection through the combination of the new techniques of MIRA and LFD. The detection of known viruses suggests that this assay is highly specific, and the detection of the positive control of the plasmid and the serially diluted RNA of BCoV-positive samples suggests that this assay is almost as sensitive and specific as the RT-qPCR reported previously.

Compared with RT-qPCR assays, MIRA-LFD assays do not require expensive instruments. Compared with other isothermal amplification-based assays, such as LAMP (33, 34), MIRA-LFD assays are more easy in design. Moreover, MIRA-LFD assays require less time than RT-qPCR and LAMP assays. Notably, the collected clinical samples included both respiratory and feces samples, indicating that our MIRA-LFD assay applies to both types of samples. Therefore, MIRA-LFD assays are more suitable for on-site detection, which has advantages in taking prompt control measure or specific treatment and preventing the spread of infectious diseases.

MIRA-LFD assays have been employed in the detection of some human pathogens, such as SARS-CoV-2 and hepatitis C virus (35–37), but they have been rarely employed in the veterinary field. To our knowledge, this is the first study to report the detection of BCoV using this method, and this study shall inspire the development of MIRA-LFD assays for the detection of other human and animal viruses.

In conclusion, we developed a novel, specific, and sensitive MIRA-LFD assay targeting the viral N gene for the rapid and on-site detection of BCoVs, which can be applied for the diagnosis, surveillance, and control of BCoV infections in cattle and other animals as well as in humans.

## Data availability statement

The original contributions presented in the study are included in the article/supplementary material, further inquiries can be directed to the corresponding author.

## Author contributions

HY and ZP contributed to the design of the experimental work and revised the manuscript. CJ contributed to the experimental work and drafted the manuscript. YF, QG, and YZ performed the statistical analysis. JM and RS revised the manuscript. All authors reviewed the manuscript.
